# BraNet: a mobil application for breast image classification based on deep learning algorithms

**DOI:** 10.1007/s11517-024-03084-1

**Published:** 2024-05-02

**Authors:** Yuliana Jiménez-Gaona, María José Rodríguez Álvarez, Darwin Castillo-Malla, Santiago García-Jaen, Diana Carrión-Figueroa, Patricio Corral-Domínguez, Vasudevan Lakshminarayanan

**Affiliations:** 1 Departamento de Química y Ciencias Exactas, Universidad Técnica Particular de Loja, San Cayetano Alto s/n CP1101608, Loja, Ecuador; 2 Instituto de Instrumentación para la Imagen Molecular I3M, Universitat Politécnica de Valencia, 46022 Valencia, Spain; 3 Theoretical and Experimental Epistemology Lab, School of Opto ΩN2L3G1, Waterloo, Canada; 4 Hospital-IESS del Sur de Quito, Av. 18 de Septiembre, Quito, Ecuador; 5 Corporación Médica Monte Sinaí-CIPAM (Centro Integral de Patología Mamaria) Cuenca-Ecuador, Facultad de Ciencias Médicas, Universidad de Cuenca, Cuenca, 010203 Ecuador; 6 Department of Systems Design Engineering, Physics, and Electrical and Computer Engineering, University of Waterloo, Waterloo, ON N2L3G1 Canada

**Keywords:** Breast cancer, Mobil app, Deep learning, Ultrasound, Mammography

## Abstract

Mobile health apps are widely used for breast cancer detection using artificial intelligence algorithms, providing radiologists with second opinions and reducing false diagnoses. This study aims to develop an open-source mobile app named “BraNet” for 2D breast imaging segmentation and classification using deep learning algorithms. During the phase off-line, an SNGAN model was previously trained for synthetic image generation, and subsequently, these images were used to pre-trained SAM and ResNet18 segmentation and classification models. During phase online, the BraNet app was developed using the react native framework, offering a modular deep-learning pipeline for mammography (DM) and ultrasound (US) breast imaging classification. This application operates on a client–server architecture and was implemented in Python for iOS and Android devices. Then, two diagnostic radiologists were given a reading test of 290 total original RoI images to assign the perceived breast tissue type. The reader’s agreement was assessed using the kappa coefficient. The BraNet App Mobil exhibited the highest accuracy in benign and malignant US images (94.7%/93.6%) classification compared to DM during training I (80.9%/76.9%) and training II (73.7/72.3%). The information contrasts with radiological experts’ accuracy, with DM classification being 29%, concerning US 70% for both readers, because they achieved a higher accuracy in US ROI classification than DM images. The kappa value indicates a fair agreement (0.3) for DM images and moderate agreement (0.4) for US images in both readers. It means that not only the amount of data is essential in training deep learning algorithms. Also, it is vital to consider the variety of abnormalities, especially in the mammography data, where several BI-RADS categories are present (microcalcifications, nodules, mass, asymmetry, and dense breasts) and can affect the API accuracy model.

**Graphical abstract:**

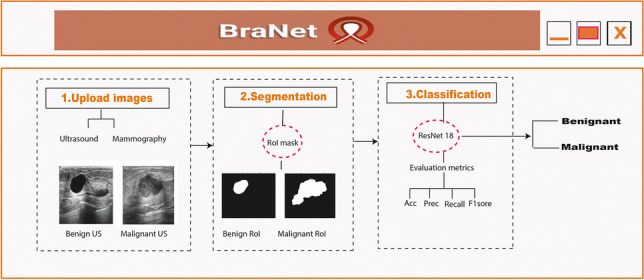

**Supplementary Information:**

The online version contains supplementary material available at 10.1007/s11517-024-03084-1.

## Introduction

Today, in the healthcare landscape, artificial intelligence tools hold great promise for clinicians by enhancing breast cancer diagnostics and tailoring treatment strategies to match the disease’s characteristics [1–3]. However, in the same line, there are some alternatives, such as command-line tools with shell scripts [4] and manual, semi-automated, and fully automated methods for image processing [5]; these options are not user-friendly for specialists and researchers without a background in computer science. Furthermore, the available graphical interface tools are often task-specific [6, 7], focusing on contour delineation, segmentation, or classification.

In this context, radiomics constitutes an emerging field in medical imaging and offers the potential to extract diagnostic and prognostic information from 2D grayscale images by analyzing lesion features [1, 2]. Hence, graphical and mobile tools are elevating the role of radiomics in biomedical research, potentially serving as a second opinion for radiologists in breast lesion detection. Specifically, computer-aided diagnosis (CAD) systems based on deep/machine learning (DL/ML) play a crucial role in addressing various computer vision challenges, such as medical image pre-processing with super-resolution [8–11] and denoising, data augmentation [12–15], medical image segmentation [16–18] (e.g., NiftyNet [6], MIScnn [16], and NiftySeg [17]), image classification [19], computer-assisted interventions [5], image recognition [20], and annotation [5].

In the context of detecting cancer, there are several radiomic projects, CAD based on deep/machine learning (DL/ML) systems, and studies that propose different artificial intelligence techniques that help to provide decision support for many applications in the patient care processes, such as lesion detection, characterization, cancer staging, and treatment planning. The major challenge in this field of research is to build a fully automatic CAD system that can analyze large quantities of images to provide an accurate diagnosis and, at the same time, robust enough to handle the biological variations in humans [21].

The most successful DL algorithms used in the processing of medical images are convolutional neural networks (CNNs), generative adversarial networks (GANs), and recurrent neural networks (RNNs), which play a crucial role in improving healthcare outcomes by providing accurate and efficient analysis in processing medical images, each offering unique capabilities in data augmentation, pattern recognition, and feature extraction [22]. In the early detection of breast cancer, CAD systems have several stages: (i) image collection, (ii) annotation and detection of tumors based on the region of interest (ROI), (iii) segmentation, (iv) classification based on the ROI shape using deep learning models, and (v) performance evaluation of the models [23, 24].

Image collection and annotation are the main challenges in performing large-scale medical image analysis using DL algorithms. Some CNNs-based options to consider as mask segmentation for detected tumors in medical images are you only look once (YOLO) [25], region-based convolutional neural network (R-CNN) [26, 27] and their variants (Mask R-CNN [26] and Faster R-CNN [27]), deep neural networks such as natural language process (NLP), which can help us to automatically identify and extract relevant information from radiology clinical reports and images [28].

Although there is a variety of CAD systems developed concerning breast cancer, it is also important to mention that there are systems deployed in mobile applications for use in the smartphone, e.g., in [29], an automated breast cancer diagnosis system on mobile phones for taking photos of ultrasound reports was implemented. The authors include the automatic extraction of intricate image features by convolutional neural networks (CNNs) and the precise classification of breast masses. It eliminates the need for manual feature engineering and reduces human error. These applications streamline the diagnostic process, increase efficiency, and, most importantly, enhance patient outcomes by providing reliable, consistent, and accessible early breast cancer detection and treatment tools.

## Related work

In this section, we will briefly introduce NLP and CNN modeling as more recent approaches using neural networks and discuss how several authors have used these models in radiomics and biomedical applications.

One of the main fundamentals of NLP is extracting image information using patterns such as the accession number, series number, and image number. Information about the imaging modality, magnetic resonance imaging (MRI), CT (computed tomography), positron emission tomography (PET), ultrasound (US), and mammography imaging can be relevant, too. It can be extracted from the accession number and image number, where the patient identification number (ID) can be appropriate if the patient’s history is of interest.

Linna et al. [30] indicate that NLP tools in radiology and other medical settings have been used for information retrieval and classification. NLP-based algorithms have opened more possibilities for medical image processing, detecting findings, and giving possible diagnoses [31]. Wang et al. [32] suggested that cancers are the most common subject area in NLP-assisted medical research on diseases, with breast cancers (23.30%) and lung cancers (14.56%) with the highest proportion of studies. Also, Luo et al. [33] specified that NLP is useful for creating new automated tools that could improve clinical workflows and unlock unstructured textual information contained in radiology and clinical reports for the development of radiology and clinical artificial intelligence applications.

Prabadevi et al. [34] proposed a machine learning system using WEKA algorithms to detect cancer staging classification. Buckley et al. [35] used NLP to extract clinical information from > 76,000 breast pathology reports, the model of which demonstrated a sensitivity and specificity of 99.1% and 96.5% compared to expert humans. Chen et al. [36] proposed an NLP extraction pipeline system that accepts scanned images of operative and pathology reports. The system achieved 91.9% (operative) and 95.4% (pathology) accuracy. The pipeline accurately extracted outcomes data pertinent to breast cancer tumor characteristics, prognostic factors, and treatment-related variables. Liu et al. [37] implemented an NLP program to extract index lesions and their corresponding imaging features accurately from the text of breast MRI reports.

The NLP system demonstrated 91% recall and 99.6% precision in correctly identifying and extracting image features from the index lesions. The recall and precision for correctly identifying the BI-RADS categories were 96.6% and 94.8%, respectively. Kirillov et al. [38] created the NLP-based segment anything model (SAM) as a mask extraction and promptable segmentation task. Thus, it can transfer zero-shot [39] to new image distributions.

The NLP system demonstrated 91% recall and 99.6% precision in correctly identifying and extracting image features from the index lesions. The recall and precision for correctly identifying the BI-RADS categories were 96.6% and 94.8%, respectively. Kirillov et al. [38] created the NLP-based segment anything model (SAM) as a mask extraction and promptable segmentation task. Thus, it can transfer zero-shot [39] to new image distributions.

Likewise, in a CAD system, the classification task is an important step after the segmentation process. The most widely used deep learning-based algorithms for image classification are CNN models (ResNet [40], DenseNet [41], NasNet [42, 43], VGG-16 [44], GoogLeNet [45], and Inception-V3 [46]).

Several authors [47–51] have used models for benign and malignant breast mass classification. The CNN used for breast classification is divided into two main categories: (i) novo-trained model (e.g., Scratch) and (ii) transfer learning-based models that exploited previously trained networks (e.g., AlexNet, VGG-Net, GoogLeNet, and ResNet) [47]. In [48], the ResNet model was used as a classification training model using an original and synthetic mammography (DDSM) dataset, obtaining a performance of 67.6 and 72.5%, respectively.

In [49], several CNN models were proposed (GoogLeNet, Visual Geometry Group Network (VGGNet), and ResNet), to classify malignant and benign cells using average pooling classification. The results overcome all the other deep learning architectures in terms of accuracy (97.67%). However, the choice of architecture depends on the specific problem and involves commitments between factors such as model size, computational efficiency, and accuracy.

Despite the extensive availability of medical radiomic tool research and CAD-based deep learning systems [52, 53], this technology has limited support within mobile app infrastructure for 2D breast medical image analysis. Consequently, the BraNet’s workflow has two main phases off-line and on-line, to achieve the following aims: (i) to develop a mobile app based on deep learning models for segmenting and classifying 2D breast images into benign and malignant lesions and (ii) to implement statistical metrics as a prediction performance evaluation tool.

## Methods

### Data collection

We collected seven open-access breast image databases, including three datasets of breast ultrasound (US) images and four datasets of mammography images.

(i) Breast Ultrasound Images Dataset (BUSI): this dataset, gathered by [43], comprises 780 images (133 normal, 437 benign, and 210 malignant).
(ii) Dataset A: collected by Rodrigues et al. [54] available at (https://data.mendeley.com/datasets/wmy84gzngw/1), Dataset A contains 250 breast US images (100 benign and 150 malignant).
(iii) Dataset B: Comprising 163 US images, these data were acquired from the UDIAT Diagnostic Centre of the Parc Tauli Corporation, Sabadell, Spain [55].
(iv) CBIS-DDSM: Curated Breast Imaging Subset–Digital Database for Screening Mammography, accessible at (https://n9.cl/qtl48), this database comprises 2620 cases [56].
(v) mini-MIAS (Mammographic Image Analysis Society): available at http://peipa.essex.ac.uk/info/mias.html), includes 322 (208 normal, 63 benign and 51 malignant images) Medio Lateral Oblique (MLO) mammograms from 161 patients [57, 58].
(vi) Inbreast: this dataset comprises a total of 115 images and can be found at (https://biokeanos.com/source/INBreast) [59].
(vii) VinDr-Mammo: introduces a large-scale full-field digital mammography dataset of 5,000 four-view exams (https://physionet.org/content/vindr-mammo/1.0.0/) [60].

### Pretraining models in phase off-line

#### Data normalization and automatic ROI annotation

An ROI annotation is needed from a large dataset of US and mammography images from the above public database to improve the previously trained GAN and ResNet models and their computational performance. The breast images vary in size, see Table 1.

**Table 1:** US and mammography ROIs

Type	Training	Database	Image size (pixels)	Benignant	Malignant
**US**	I	BUSI	500 × 500	427	201
		Dataset B	-	100	150
		BUS (UDIAT)	760 × 570	109	54
		**Total 1041**		636	415
**DM**	I	Mini-MIAS	1024 × 1024	118	91
		INbreast	3328 × 4084	2106	144
			256 × 3328		
		CBIS-DDSM	3784 × 5912	1225	779
	II	VinDr-Mammo	3518 × 2800	893	1236
		**Total 5892**		4342	1550

Thus, it is necessary to perform transformations and standardize the images taken at different sizes to a single dimension (128 × 128 × 1 pixel). It was also necessary to transform it to a single channel (grayscale pixel) and normalize it in the range [− 1,1] with a mean of 0.5 and a standard deviation of 0.5. The torch-vision (pytorch) library and Jupyter notebook algorithm (crop_vindr_images.ipynb) were used as the image annotation region processes to identify ROIs that may contain lesions.

**Fig. 1:**
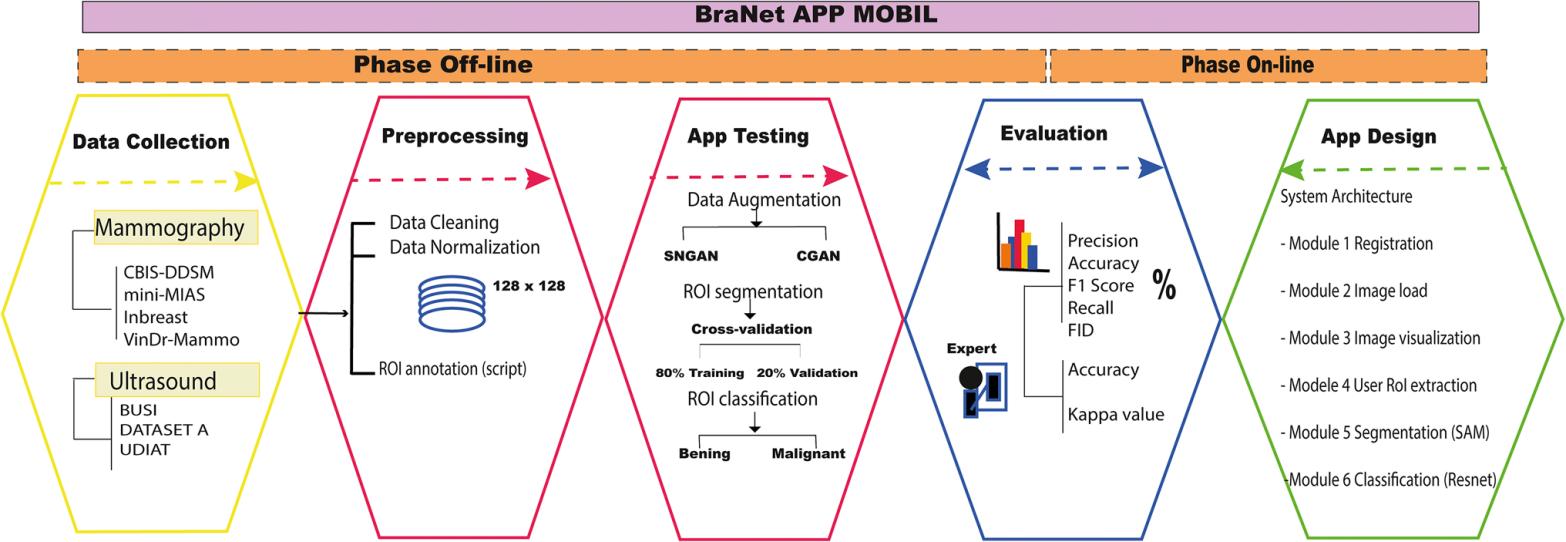
The BraNet’s workflow has two main phases off-line: (i) breast data collection from public databases and (ii) data preparation and app testing and evaluation. On-line: (iii) system architecture design and (iv) statistical metrics comparison between the mobile application and human experts

Figure 1 details the overall process followed in this study.

#### User ROI extraction and segmentation

As other studies have pointed out [61], to improve the detection accuracy, smaller patches (i.e., RoIs) where all breast masses and micros (e.g., cysts and calcification) are included inside this extracted area are generated from the original mammogram. In most mammogram images, 32 to 56% are background pixels, which do not contribute to breast cancer diagnosis.

In this research, the segment anything model (SAM) [38] (an encoder-decoder architecture based on NLP prompt-based learning) [35] was trained as automatic ROI segmentation before being implemented in the Module 5 (BraNet application phase on-line). SAM is an open-source software, and the quality of the segmentation masks was rigorously previously evaluated, with automatic masks deemed high quality and effective for training models, leading to the decision to include automatically generated masks.

NLP tasks include sentence boundary detection, tokenization, and problem-specific segmentations, and the *SamAutomaticMaskGenerato* function was used for automatic mask extraction [28]. SAM model is available under a permissive open license (Apache 2.0) at https://segment-anything.com.

The SAM predefined hyperparameters used are as follows: points_per_side (32), points_per_batch (64), pred_iou_thresh (0.88), stability_score_thresh (0.95), stability_score_offset (1.0), box_nms_thresh (0.7), crop_n_layers (0), crop_nms_thresh (0.7), crop_overlap_ratio (512/1500), crop_n_points_downscale_factor (1), point_grids (Null), min_mask_region_area (0), and output_mode (‘binary_mask’). Also, SAM accuracy ROI segmentation is evaluated by the intersection-over-union (Jaccard index) metric [18] using *calculate_stability_score* function.

The model was trained on a large and diverse set of masks of mammography and US images. These ROIs were previously extracted from the Mini-MIAS, Inbreast, and VinDr-Mammo databases (corresponding annotated bounding boxes are available in a.csv file). However, it is worth noting that its authors had already classified the ROIs from the CBIS-DDSM dataset; thus, no additional ROI extraction was required. Regarding US images, RoIs were extracted from the BUSI and Dataset B (UDIAT), excluding Dataset A, because it already contained RoIs.

A total of 6592 breast ROI images were used for pre-trained SAM and GAN models, with 4463 mammography images (benign and malignant) and 1041 US images, as shown in Table 1.

#### Data augmentation

This technique was previously employed in the phase off-line to mitigate the risk of overfitting effectively. To generate new realistic images and improve BraNet’s classification task performance, all ROIs were previously augmented by a GAN using the spectral normalization technique (SNGAN). SNGAN introduces a novel weight normalization technique known as spectral normalization to enhance the training stability of the discriminator network [62, 63], serving as the foundation for synthetic image generation, which use Hinge loss function (see Eq. 1).

1 $${L}_{D}=\begin{array}{c}-{{\text{E}}}_{\left({\text{x}}, {\text{y}}\right)\sim {{\text{P}}}_{{\text{data}}}}\left[{\text{min}}\left(0,-1+{\text{D}}\left({\text{x}},{\text{y}}\right)\right)\right]-{{\text{E}}}_{{\text{z}}\sim {{\text{P}}}_{{\text{z}},{\text{y}}}{{\text{P}}}_{{\text{data}}}}\left[{\text{min}}\left(0,-1+{\text{D}}\left({\text{G}}\left({\text{z}}\right),{\text{y}}\right)\right)\right] {{\text{L}}}_{{\text{G}}}\\ =-{{\text{E}}}_{{\text{z}}\sim {{\text{P}}}_{{\text{z}},{\text{y}}}{{\text{P}}}_{{\text{data}}}}{\text{D}}\left({\text{G}}\left({\text{z}}\right),{\text{y}}\right)\end{array}$$

where ${P}_{{\text{data}}}$ is the real data distribution, $P(z)$ is a prior distribution on noise vector *z*, $D\left(x\right)$ denotes the probability that *x* comes from the real data rather than generated data, ${E}_{x\sim {P}_{{\text{data}}}}$ represents the expectation of *x* from real data distribution ${P}_{{\text{data}}}$, and ${E}_{z\sim {P}_{(z)}}$ is the expectation of *z* sampled from noise.

The *clean-fid* library was used to obtain the FID value, using the Tensorflow and PyTorch libraries, some original implementations of the metric were taken from Parmar et al. [64]. The GAN model was trained using a cross-validation technique in Google Colab Pro 1 GPU model V100 with CUDA cores execution; with the hyperparameters detailed in Table 2.

**Table 2:** Hyperparameter tunning of deep learning models

Hyperparameter	SNGAN	ResNet
	DM/US	DM/US
Batch size	64/32	32/16
Image size	128 × 128	128 × 128
Nro epochs	100	100
Learning rate	2 × 10^–4^/1.5 × 10^–4^	2 × 10^–5^
Optimizer	Adam	Adam
Activation function	LeakyReLU	ReLu
β1	0.3	0.1
β2	0.999/0.75	0.9
Latent vector	100	-
Loss function	Hinge/BCE	2.190
Optimization function (discriminator)	LeakyReLU	-
Optimization function (generator)	ReLU	-

#### Cross-validation analysis

The technique divides the dataset into multiple folds and trains (DM: training I (4463) and training II (6592) and US: training I (1041)) the model on different subsets while validating the remaining fold can provide a more robust estimate of the model's performance, effectively mitigating the risk of overfitting. It helps detect overfitting early and tune the model accordingly. A total of 6933 benign and malignant ROIs were split into 80% training and 20% validation, using the Sklearn library from Pytorch, see Table 3.

**Table 3:** Training and validation datasets of DM 80% (5273) and 20% (1319) and US 80% (832) and 20% (209) breast images

Classes	Training datasets		Validation dataset	
Image type	US	DM	US	DM
Bening	505	3471	131	871
Malignant	327	1802	78	448
Total	**832**	**5273**	**209**	**1319**

#### ROI classification process

Before implementing Module 6 (ROI image classification) in the BraNet mobil interface, the ResNet model was pre-trained on a large set of generated mammography and US ROIs using also cross-validation technique.

##### ResNet18 training model

The ResNet18 CNN-deep learning-based classification model has been widely used in medical image classification, especially in breast lesion diagnosis and detection, and was chosen for its effectiveness in transfer learning, offering reduced training time and the automatic extraction of features [40]. This approach effectively mitigates the issues of vanishing or exploding gradients that can arise from increasing neural network depth, ultimately leading to improved accuracy [65–68].

Thus, to train the ResNet model and distinguish between malignant and benign breast lesions, the datasets were divided in two categories: (i) dataset A (original + synthetic ROIs) and (ii) dataset B (synthetic ROIs). The model consists of three convolutional layers and two fully connected layers. The kernel size for the first convolutional layer is 5 × 5, and, for the rest, 3 × 3. The size of the first and the second fully connected layers are 128 and 2 (the number of classes), with a dropout of 0.5. After the flattening and the first fully connected layers, the ReLU activation function for all layers except the output layer, where softmax was used. The model was pre-trained with the PyTorch library using a Google Colab Pro 1 GPU model V100 with CUDA cores execution; the training hyperparameters are outlined in Table 2.

### System architecture in phase on-line

The system architecture consists of two primary components facilitating scalability and system maintenance: (i) the mobile application and (ii) the backend server, following a client–server architecture.

**Fig. 2:**
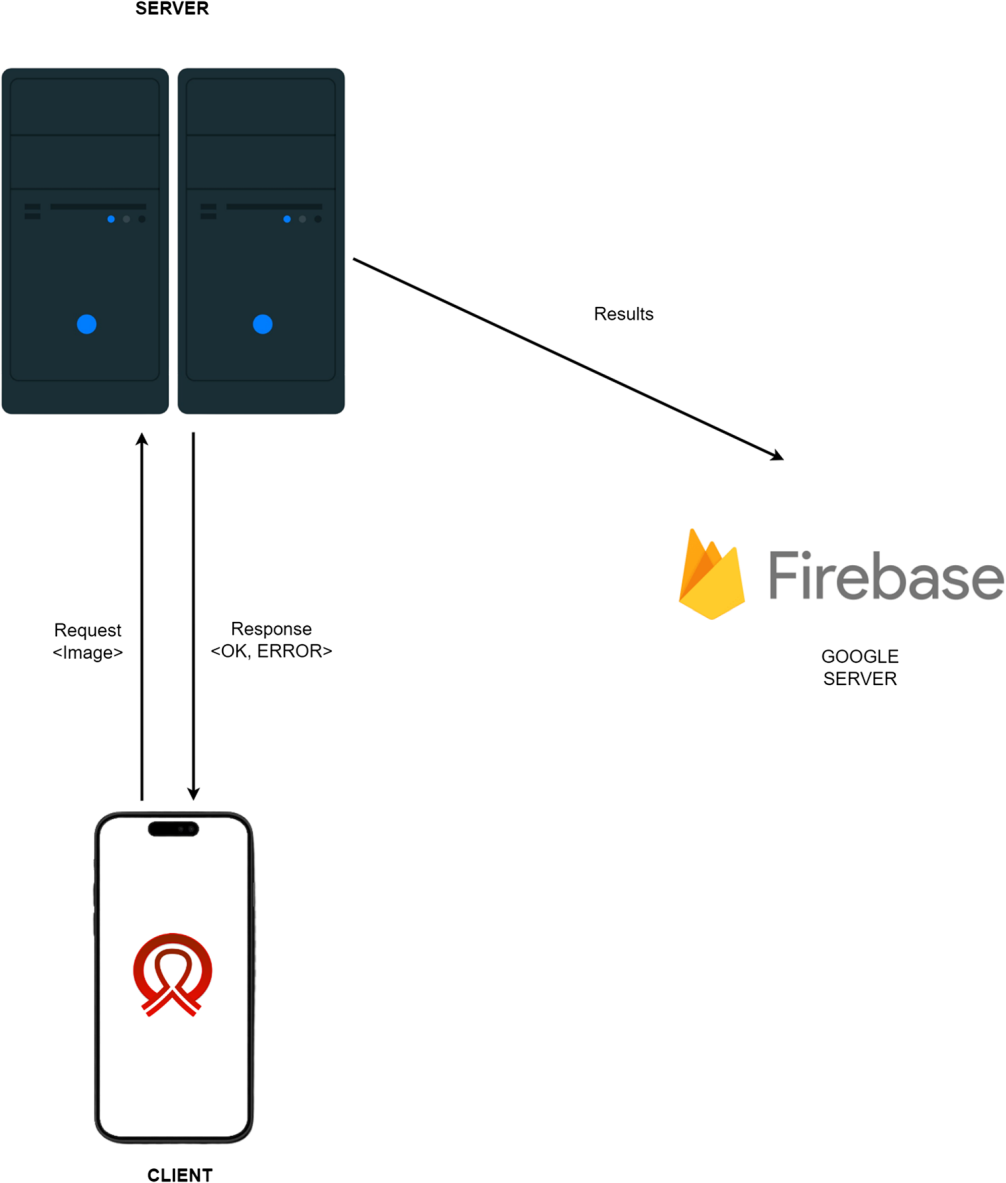
Client–server architecture to BraNet App

The mobile application is a client that communicates with the backend server to request services and image analysis. The backend server processes these requests and returns the results to the client for display (see Fig. 2). The backend server was developed using react native and was implemented in the Python programming language.

The mobile application comprises several interrelated components:

- Module 1: Registration, Login Synchronization, and User Profile Information. Data generated by the application, such as images and metadata, is stored in Firebase (a mobile and web application development platform). Firebase is also used for user authentication, mobile application registration, and log in.
- Module 2: Image Import allows users to upload breast ultrasound and mammography images in PNG, JPG, and JPEG formats, with a maximum file size of 10 MB.
- Module 3: Visualization Area (a history of image analysis results, image analysis capabilities, and user assistance).
- Module 4: User ROI extraction.
- Module 5: ROI segmentation (see Section 2.2.2).
- Module 6: ROI Image Classification (see Section 2.2.4).

### Evaluation metrics in phase off-line and on-line

#### Quality of synthetic image

The FID and KID quantitative feature-based metrics have been applied to evaluate the quality of real and synthesized ROIs generated by GANs models and to compute the distance between the vector representation of the synthesized and authentic images.

*Fréchet inception distance (FID)*: FID compares the distributions of the original and synthetic images to assess how well the generated images represent the training dataset. Lower FID scores indicate better quality images [69], and it is calculated as shown in Eq. 2:

2 $${\text{FID}}={\Vert {\mu }_{r}-{\mu }_{g}\Vert }^{2}+Tr({\sum }_{r}+{\sum }_{g}-2{({\sum }_{r}{\sum }_{g})}^{1/2})$$

Here, *µ*_*r*_ represents the mean of the feature vector calculated from the real images, *µ*_*g*_ is the mean of the feature vector calculated from the fake images, Σ_r_ is the covariance of the feature vector from the real images, and *Σ*_*g*_ is the covariance of the feature vector from the fake images.

##### Kernel inception distance (KID)

KID employs the cubic kernel to compare the skewness, mean, and variance [69]. A lower KID value signifies a higher visual similarity between the actual and generated images. The cubic polynomial kernel is defined as shown in Eq. 3):

3 $$k\left(x,y\right)={\left(\frac{1}{d} {x}^{T}y+1\right)}^{3}$$

where* d* represents the dimension of the feature space for vectors *x* and *y*.

#### BraNet’s classification performance evaluation

For assessing the *BraNet’s* classifier’s performance, we employed a confusion matrix, *F*1 score (Dice), accuracy (Acc), precision (Prec), sensitivity (Sen), recall, and specificity (Spec) [24] metrics (see Tables 4 and 5). The accuracy of the model was calculated using statistical score libraries such as the classification report and confusion matrix from the Python sci-kit-learn module.

**Table 4:** Confusion matrix to distinguish between two classes (benign, and malignant)

Actual classes	Classes	Predicted classes		Measures
		Positive	Negative	
		C_1_ (benign)	C_2_ (malignant)	
	C_1_ (benign)	TP	FP	PPV
	C_2_ (malignant)	FN	TN	NPV
	Measures	Sen	Spec	Acc

**Table 5:** Validation assessment metrics

Model	Equation
Accuracy	${\text{Acc}}=\left(\frac{{\text{TP}}+{\text{TN}}}{{\text{TP}}+{\text{TN}}+{\text{FP}}+{\text{FN}}}\right)$
Sensitivity	${\text{Sen}}=\left(\frac{{\text{TP}}}{{\text{TP}}+{\text{FN}}}\right)$
Specificity	${\text{Spec}}=\left(\frac{{\text{TN}}}{{\text{TN}}+{\text{FP}}}\right)$
Precision	${\text{Prec}}=\left(\frac{{\text{TP}}}{{\text{TP}}+{\text{FP}}}\right)$
*F*1 score	$F1\mathrm{\;score}=2\times\left(\frac{\mathrm{Prec\;}\times\mathrm{\;recall}}{\text{prec }+\text{recall}}\right)$

#### Human expert evaluation

Two senior radiologists were asked to assess, annotate, and classify images independently to ensure that BI-RADS categories are correctly assigned. Representative original ROI images for each breast type is available in https://drive.google.com/drive/folders/1HMeqPfI8qL58hAqwVpZupH6uq4W_kHrI.

A comparison between the images tested by human experts and those annotated in public databases was conducted using an independent test set of 212 mammography images (47 malignant and 165 non-malignant) and 78 US images (24 malignant and 54 non-malignant).

Two diagnostic radiologists (reader 1 with 20 years of experience and reader 2 with 13 years of experience) were given a reading test consisting of 290 total original RoI images to assign the perceived breast tissue type. The readers rated each image as (1) benign or (0) malignant.

##### Kappa coefficient and overall accuracy

Furthermore, the agreement between the two readers’ answers (considering all elements of error matrix) was assessed by determining the kappa coefficient (*K*), using the ranges between 0 (when there is no agreement) and 1 (when there is substantial agreement), and is calculated using the Eq. 4. The error matrix was calculated by comparing the two readers’ choices from five possibilities and was interpreted as follows: < 0.2 slight; 0.21–0.40 fair; 0.41–0.60 moderate; 0.61–0.80 high; and 0.81–1.0 almost perfect [70].

4 $$k=\frac{{P}_{0}-{P}_{e}}{1-{P}_{e}}$$

*P*_o_ is the correctly allocated samples (agreement cases), and *P*_e_ is the hypothetical random agreement.

The overall accuracy (Eq. 5) allows the description of model performance and is calculated by dividing the total number of correctly classified samples by the total number of samples.

5 $${\text{Acc}}= \frac{{C}_{s}}{{N}_{s}}$$

*C*_s_ is the number of correct samples classified, and *N*_s_ is the total number of samples.

## Results

The main results are categorized into two phases, off-line and on-line. First, we introduced the preprocessing and pre-training models’ section with data augmentation (GAN), segmentation (SAM), and classification (ResNet) algorithms. Then, we presented the on-line phase with a practical utility of BraNet’s user interface and its modules.

### Preprocessing and pretrained models

#### Synthetic data to feed the classification network

A significantly number of synthetic RoIs (10,000 (training I) and 2000 (training II) mammography RoIs and 4000 US RoIs (training I)) were generated by SNGAN to feed the classifier. The loss function and accuracy of the generator and discriminator play a crucial role in assessing the training stability and performance of GANs. A stable GAN is characterized by a discriminator loss around 0.5 or higher than 0.7, while the generator loss typically ranges from 1.0 to higher values like 1.5, 2.0, or even more. The accuracy of the discriminator, both on real and synthetic images, is expected to hover around 70 to 80%. Appendix Table 9 presents the accuracy plot to SNGAN.

The average FID and KID values in SNGAN are 58.80/0.052 and 52.34/0.051 for mammography training I and training II, respectively, and 116.85/0.06 for the training I in US (see Appendix Fig. 6). The lowest values indicate that SNGAN-generated synthetic images closely resemble to the original mammography and US images in clinical characteristics, suggesting their potential utility in clinical data augmentation and training, particularly for enhancing diagnostic skills in breast imaging.

#### ResNet training model

The model shows the highest accuracy in US image classification (see Table 6) concerning the mammography dataset. Although the network received more mammography images (6592) as input (Mini-MIAS, Inbreast, CBIS-DDSM, and VinDr-Mammo) with respect to the small number (1041) of US data (BUSI, UDIAT, and DATASET A). It means that not only the amount of the data is important to train deep learning algorithms. Also, it is important to considerer the variety of abnormalities especially in the mammography data, such as microcalcifications, nodules, mass, asymmetry, and dense breasts, because it can improve the accuracy of the ResNet training model.

**Table 6:** ResNet statistical performance evaluation in US and DM image classification

RESNET18	Image modality					
	Training I (1041)		Training I (4463)		Training II (6592)	
Image type	US (%)		DM (%)		DMb(%)	
Classes	**Benignant**	**Malignant**	**Benignant**	**Malignant**	**Benignant**	**Malignant**
Accuracy	94.7	93.6	80.9	76.9	73.7	72.3
Precision	97	89	92	56	84	59
Recall	93	95	81	77	74	72
*F*1 score	95	92	86	65	78	65

**Fig. 3:**
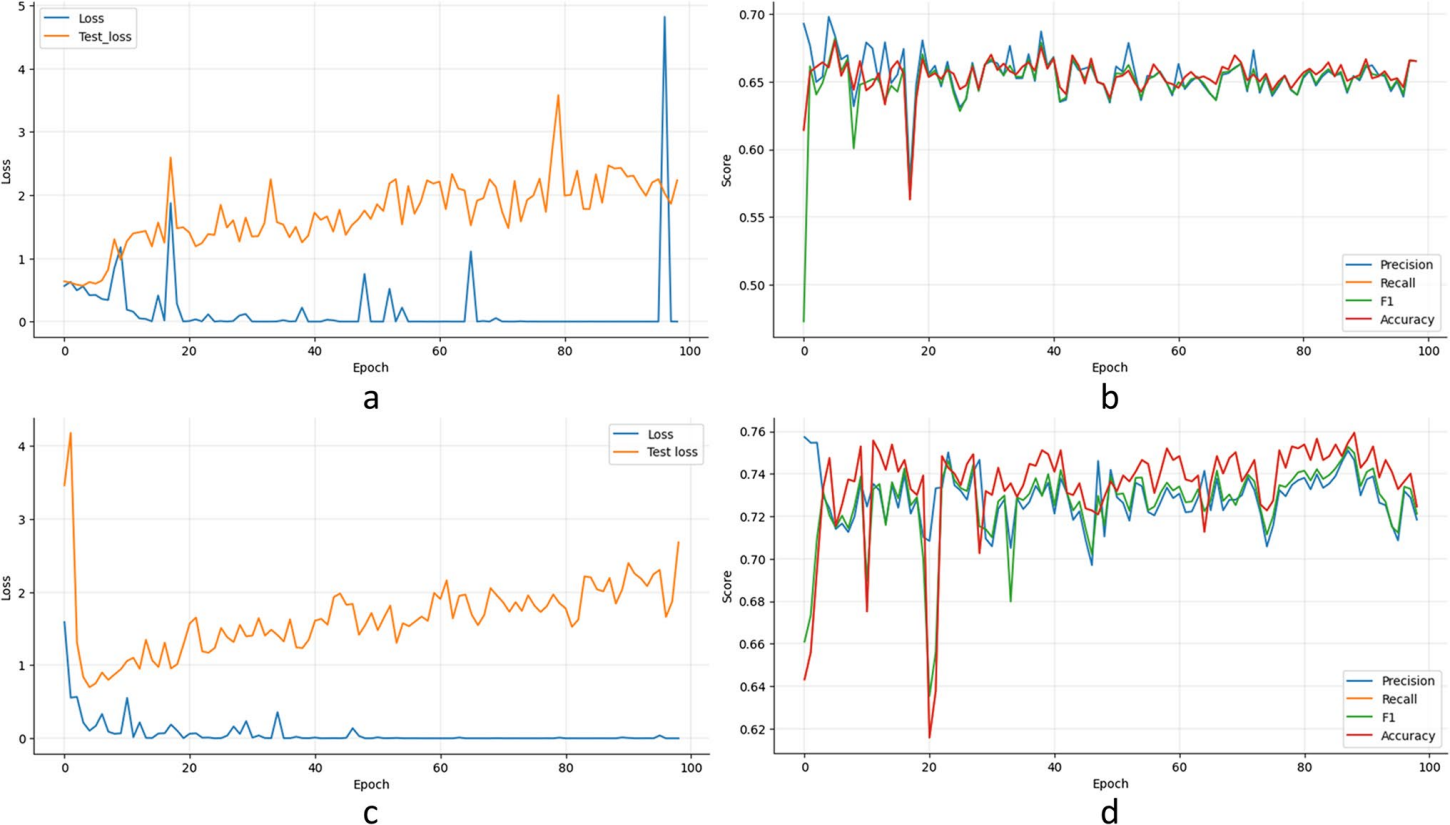
Training II and testing plots for mammography images: **a** loss vs. acc (real ROI data), **b** loss vs. acc (real + data augmentation), **c** evaluation metrics (real ROI data), and **d** evaluation metrics (real + data augmentation)

Therefore, it is essential to monitor the evolution and performance of the models using training and validation datasets, see Fig. 3a–d. Figure 3a, c displays the loss and accuracy values concerning each epoch during the ResNet training and validation model using mammography and US images, while Fig. 3b, d shows the accuracy, *F*1 score, recall, and precision by each epoch in both image types. Appendix Table 10 shows the details of the network training and validation dataset.

The BraNet App Mobil exhibited the highest accuracy in benign and malignant US images (94.7%/93.6%) classification compared to DM during training I (80.9%/76.9%) and training II (73.7/72.3%). And the ResNet model does not improve the accuracy of benign and malignant ROI lesion classification during training II compared to the previous training I.

### The BraNet and its graphical user interface (GUI)

**Fig. 4:**
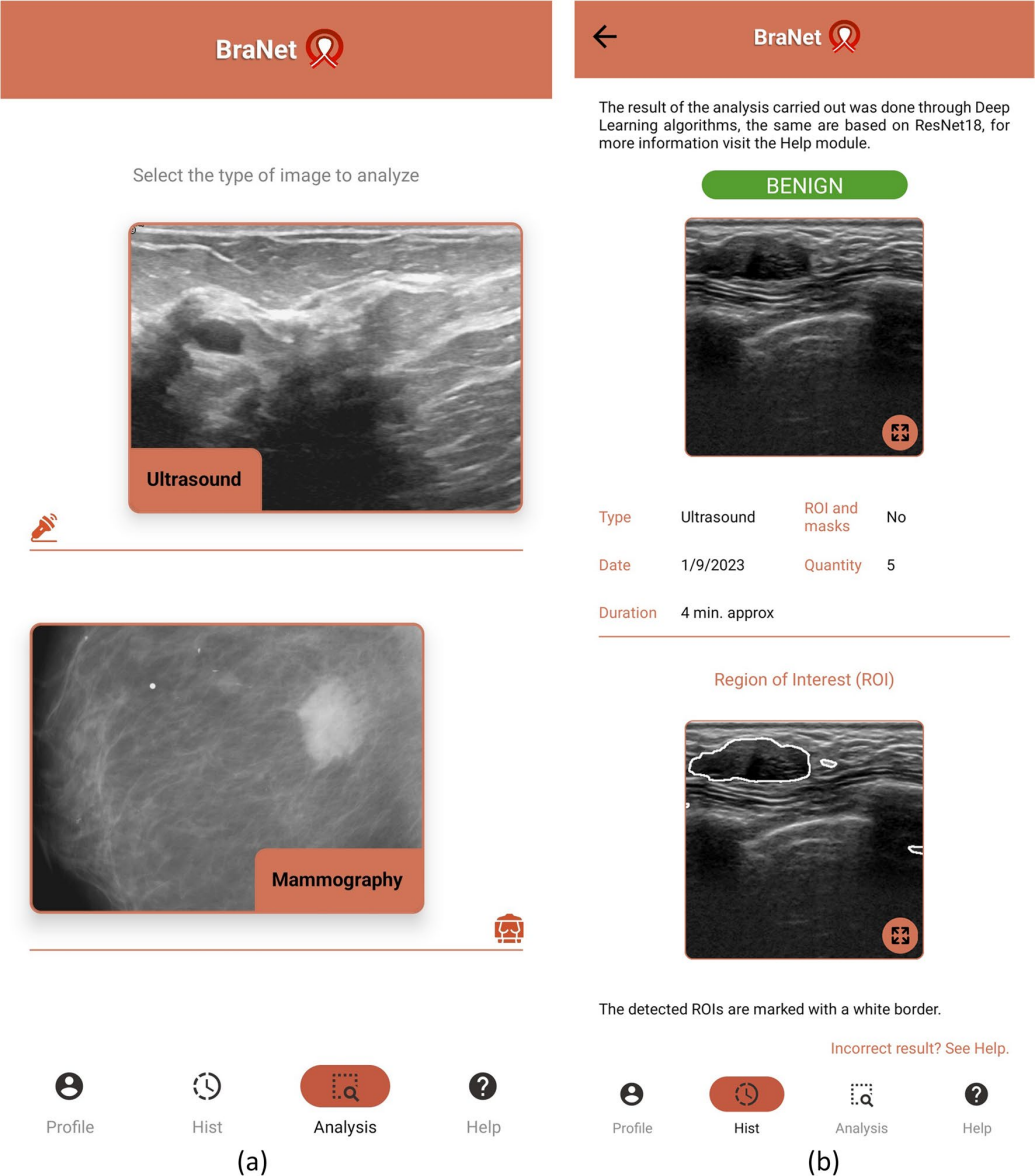
BraNet user interface within the toolbox. **a** Upload the breast image type. **b** US breast ROI selection and classification as benign class

The mobile app’s user interface was developed using Python v3.11 and React Native as a JavaScript framework for creating native mobile applications compatible with iOS and Android platforms. The interface is composed of several modules, each serving distinct purposes:

- *Module 1:* Registration, Login Synchronization, and User Profile Information: This module handles user registration and login functionalities, synchronizing user data and providing access to user profile information.
- *Module 2:* Image Import: Users can import images in standard picture formats, such as JPG, JPEG, and PNG, with a maximum size limit of 10 MB.
- *Module 3:* Visualization Area: This area displays loaded images. The selected image is displayed in grayscale, preserving the original image's aspect ratio (see Fig. 4a).
- *Module 4:* Manual ROIs Extraction: This module allows users to manually or semi-automatically create masks and define ROIs within the selected image. Masks are represented as binary matrices with the same dimensions as the loaded mammography image, where true values indicate the ROIs. Users can customize the size and sampling method for RoIs.
- *Module 5:* ROI Segmentation: Users can segment a subset or the entire set of features from the segmentation section (as shown in Fig. 4b). Before performing calculations on the image, the user must add at least one ROI in the “Regions and Masks.”
- *Module 6*: ROI Image Classification: This module employs the ResNet18 model to classify ROI images into benign and malignant classes. Example output classes are provided in Fig. 4b.

The BraNet’s graphical user interface enhances the user experience by providing intuitive image analysis and classification tools, making it a valuable resource for medical professionals and researchers. The practical usage of the tool can be accessed via the following link: https://drive.google.com/file/d/1d1vnjQ6LqOd0fdz65eaVg791d7cFRPWO/view

#### Comparison of the BraNet with human experts’ evaluation

The accuracy percentages of correct rates between benign and malign images classification for readers 1 and 2 were 29% and 42%, respectively. The reader agreement was assessed using the kappa coefficient, which values are 70% and 71% in mammography and US classification, respectively. Table 7 indicates a fair agreement (0.3) for mammography images and moderate agreement (0.4) for ultrasound images in both readers, with a change in prevalence from the lowest in US images to the highest value in mammography images, resulting in a corresponding change in sensitivity (19.2/60) to specificity (51/84.4) percentage points. This effect was statistically significant (*p* < 0.05) for either sensitivity or specificity in both image types.

**Table 7:** Interrater reliability, Cohen’s kappa, and statistical values for 2 raters in both classes

*Method*	*DM*	*US*
*Subjects*	212	78
*Agreement %*	70	71
*Kappa*	0.294	0.426
*p-value*	< .001	< .001
*Sensitivity*	19.2%	60.0%
*Specificity*	51.6%	84.8%
*Prevalence*	69.5%	57.7%
*Accuracy*	29.0%	70.5%
*PPV*	47.5%	84.4%
*NPV*	21.9%	60.9%

## Discussion

The pressing need to transition automatic medical image classification by CAD systems from research laboratories into practical clinical applications is evident. BraNet’s aims to provide an API for setting up a breast image classification pipeline with ROI mask extraction and segmentation capabilities. The tool offers an open-source solution for processing US and mammography images, complete with statistical metrics for evaluating model performance.

There have been many published examples of AI algorithms that demonstrate excellent performance in cancer detection for screening mammography. These include several algorithms trained and evaluated on private and public data sets. Table 8 compares BraNet’s performance against other state-of-the-art medical image classification applications.

**Table 8:** Comparison of the BraNet’s performance against other deep learning applications

Author	Application name	Description	Acc/Sen/Spec/Prec /AUC (%)
**Gibson et al. **[6]	NiftyNet	A DL open-source platform used in three medical image analysis applications (MRI, CT and US), including a conditional GAN model as ultrasound image generation	88/7.5/9.1/-/-
**Pang et al. **[71]	TripleGAN	Method to perform data augmentation in breast US images and feed a CNN mode to classify breast masses	90.41/87.94/ 85.86/-
**Al-Dhabyani et al. **[43]	AlexNet + GAN (CNN)	US breast classification with data augmentation. The model examines two different methods: a CNN approach and a transfer learning (TL). The results confirm an overall enhancement using augmentation methods with TL classification methods	
	-BUSI		78/-/-/-/-
	-Dataset B		80/-/-/-/-
	-BUSI + DatasetB		84/-/-/-/-
	TL		94/-/-/-/-
	-BUSI		92/-/-/-/-
	-Dataset B		99/-/-/-/-
	-BUSI + DatasetB		
**Jiménez et al. **[72]	Radiomic tool	Colposcopy image classification combining UNET + SVM as segmentation and classification cervix abnormalities	80/70/48.8/-
**Dihge et al. **[73]	NILS	A web-based tool for noninvasive lymph node staging in breast cancer	-/90/34/-/71
**To T. et al. **[74]	DUV-WSI	DUV-WSI Deep ultraviolet (DUV) fluorescence scanning microscopy provides rapid whole-surface imaging (WSI) of breast tissues. Images are split into small patches (512 × 512), and features are extracted using a pre-trained ResNet 50 as patch classification	81.7/91.7/66.7/-/-
**Qi et al. **[29]	Deep-CAD system	The breast cancer system is deployed on mobile phones, takes a photo of the US as input, and performs diagnosis on each image. Then, the system to classify images into malignant and non-malignant using CNNs	89.34/87.31/87.49
**Ours**	BraNet	A deep learning tool for breast regions classification using mammography and US images DM (TRAINING I)	
			Acc 94.7/93.6
			Prec 97/89
			Recall 93/95
			F1 score 95/92
		DM (training II)	Acc 93.7/ 72.3
			Prec 84/ 59
			Recall 74/72
			F1 score 78/65
		US (training I)	Acc 80.9/76.9
			Prec 92/56
			Recall 81/77
			F1 score 86/ 65

However, there is a significant gap in understanding how these AI applications will perform with multimodal images in the real world when radiologists use them in clinical practice [75, 76].

The BraNet Mobile App is an open interface for classifying specific 2D breast image types using deep learning models. It is believed that this is the first system for breast cancer diagnosis deployed on mobile phones to both types of images. The API’s development comprises two main phases: (i) off-line to pre-train the deep algorithms and (ii) on-line to release the app, which includes several modules, including model selection, model extraction (by a human expert), segmentation (SAM model), model classification (ResNet18 model), and model evaluation.

During the off-line phase, the pre-trained GAN algorithm was implemented as synthetic image generation, and the image quality was evaluated by two feature-based metrics FID and KID. It is widely acknowledged that the preprocessing images, quality, and diversity of the training dataset greatly impact the training of GAN and CNN deep learning models [77–79]. The lower FID and KID values mean a higher visual similarity between the real and generated images. The results (Appendix Fig. 6) indicate that the SNGAN model is suitable for mammography and US synthetic data generation with average values of FID = 52.4/ KID = 0.051 for mammography and FID = 116.85/ KID = 0.06 for US.

With these datasets Dataset A (original + synthetic ROIs) and Dataset B (real ROIs), the classification model was trained. Table 6 and Fig. 3 show the accuracy results averaged in BraNet ROI classification are as follows: (i) training I in US (94.7 (B)/93.6 (M)) and DM (80.9 (B)/76.9 (M)) and (ii) training II in DM (73.7 (B)/72.3 (M)). The result demonstrated that ResNet model during training II with original + synthetic images (where the VirDrMammo database was added) did not improve the accuracy (73.7 / 72.3%) concerning Training I (80.9/76.9). In comparison, with radiological experts, accuracy in DM was 29% concerning with 70% in DM for both readers. These results show that both API and Readers obtained a better percentage of accuracy in classifying the ROIs of mammography images than US images.

A final comparison between BraNet and radiological experts’ evaluation demonstrates that for the all-breast image types, reader accuracy was higher with US images (75%) than with original ROI images from public databases. The reader agreement was 70% and 71% in mammography and US classification, respectively. The kappa value indicates a fair agreement (0.3) for mammography images and moderate agreement (0.4) for ultrasound images in both readers (Table 7). This can be contrasted with BraNet classification accuracy (Table 8), where the API shows the highest accuracy in US image classification (Table 6) concerning the mammography dataset. Although the network received more mammography images (5892) with respect to US (1041). It means that not only the amount of the data is important to train deep learning algorithms. Also, it is important to considerer the variety of abnormalities especially in the mammography data, where several BI-RADS categories are present (microcalcifications, nodules, mass, asymmetry, and dense breasts), and can be affect the accuracy in the ResNet training model.

According to the previous results, some limitations in implementing BraNet must be addressed in future work. One is the need to classify and characterize images based on different abnormalities, such as architectural distortion, asymmetry, mass, and microcalcification. BraNet no was trained using different breast tissue types and variations in mammography and US imaging techniques; the ROI classification process was performed only using two classes 1 (benignant according to BI-RADS 1–3) and 0 (malignant according to BI-RADS 4–6) categories. Oyelade et al. [80] indicate that is better to focus on previously classified and characterizing abnormalities into architectural distortion, asymmetry, mass, and microcalcification so that training distinctively learns the features of each abnormality. It generates sufficient images for each category before training a GAN model.

Thus, in future work, we plan to annotate the datasets with more fine-grained classes to get more targeted training in GAN and CNN models. Moving forward, we should consider pre-processing with denoising, super-resolution, improving the overall image quality and reducing blur and artifacts. Also, previous breast tissue types of classification are needed to obtain a diverse range of synthetic data, resulting in a more accurate image generation and classification process using GAN and convolutional algorithms. We must also compare our image classification with other TL models, such as Nasnet and DenseNet, to ensure we use the most effective techniques.

An updated version of the BraNet application and prospectively explore the real AI/human interaction could be implemented, which can recognize full 2D images and not only resized images of 128 × 128 pixels. The app could be used for performance and load testing to assess how the application processes many images simultaneously. It simulates an increasing number of users or requests to see how the application performs under progressively higher loads.

We implement the app as a web server and realize scalability testing; incrementally increase resources (like CPU, GPU, and memory) available to the application and measure performance improvement to determine how efficiently the application scales; make full use of available CPU/GPU cores to process images in parallel, enhancing throughput; and utilize image compression techniques to reduce the size of high-resolution breast images without losing critical details necessary for analysis.

Finally, the use of IA in medical diagnosis brings about a range of ethical considerations that must be carefully navigated to ensure that the integration of these technologies benefits patients, healthcare providers, and the broader healthcare system responsibly and equitably. It is essential to highlight ethical considerations regarding using artificial intelligence in developing CAD systems in healthcare.

The patient’s well-being is paramount, necessitating a comprehensive approach to protecting their data privacy and confidentiality [81, 82]. This project ensures patient privacy through the anonymization and coding of training image databases during the application’s first and second modules, which are also publicly available.

Another ethical consideration is the fairness of AI models [83], which requires providing equitable healthcare outcomes across various patient demographics. Thus, the developed application aims to contribute to medical service equity, particularly by facilitating pathology diagnosis in rural groups and sectors typically deprioritized in healthcare, especially in developing countries.

Finally, transparency regarding the capabilities and limitations of CAD systems is fundamental [84],ensuring that medical staff and patients know that decisions and outcomes adhere to ethical standards. In this context, the developed application is merely a test prototype that aspires to achieve the necessary maturity for use in a real healthcare setting, ensuring the requisite medical reliability.

## Conclusions

In this paper, we have introduced BraNet, a mobile app for breast image classification based on deep learning algorithms. The API enables the rapid construction of breast image classification workflows, encompassing data input/output, ROI mask extraction, segmentation, and evaluation metrics. The client–server architecture, coupled with its open interface, empowers users to customize the pipeline and swiftly establish comprehensive medical image classification setups using Python libraries and the react native framework for creating native mobile applications on iOS and Android. We have demonstrated the functionality of the BraNet app by conducting automatic cross-validation on data augmentation, ROI segmentation, and classification using public ultrasound and mammography datasets, resulting in a preclinical tool. After implementing some improvements and future updates, BraNet will facilitate the migration of medical image segmentation and classification from research laboratories to practical applications. Also, ensuring that the App complies with all regulations and standards governing data privacy and security in healthcare is essential. It is only a preclinical testing phase; thus, there is still work to be done in this area. BraNet currently offers a pipeline for breast image segmentation and classification, and it will continue to receive regular updates and extensions in the future. This data must be rigorously analyzed, reported, and often published in scientific journals to ensure its accuracy and reliability.

### Supplementary Information

Below is the link to the electronic supplementary material.

Online Resource 1 (MP4 6.19 MB)

## Figures and Tables

**Fig. 5:**
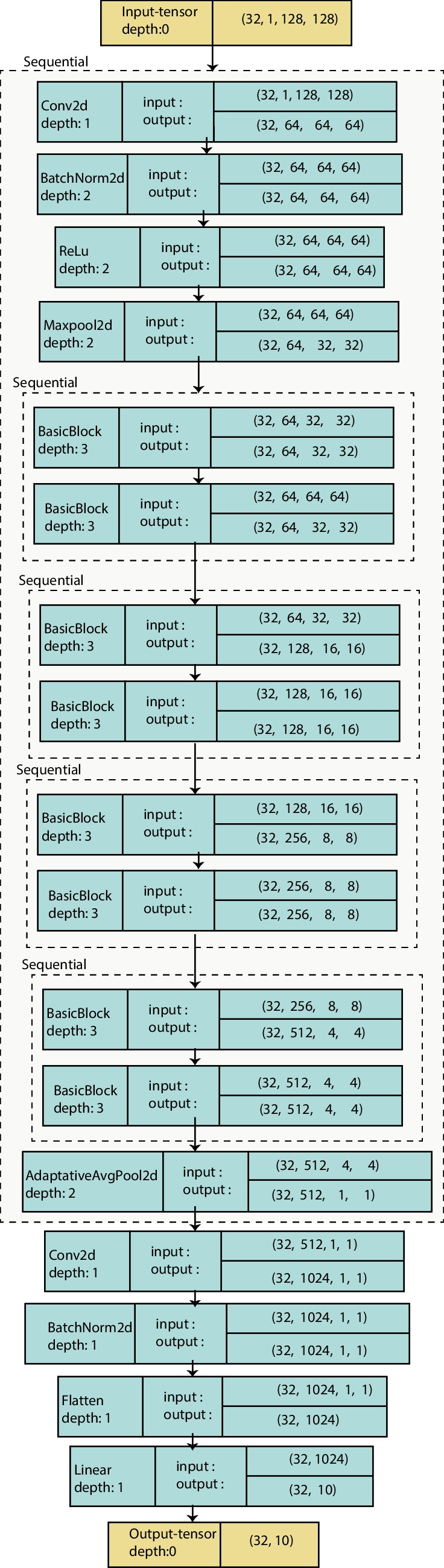
ResNet 18 classification model

**Table 9:**
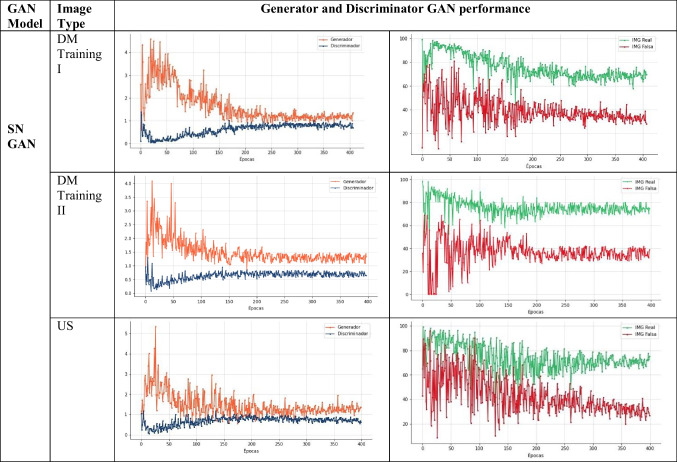
The GAN discriminator’s accuracy on real (green) and fake (red) images during training. Figures illustrate the loss and accuracy trends for SNGAN

**Fig. 6:**
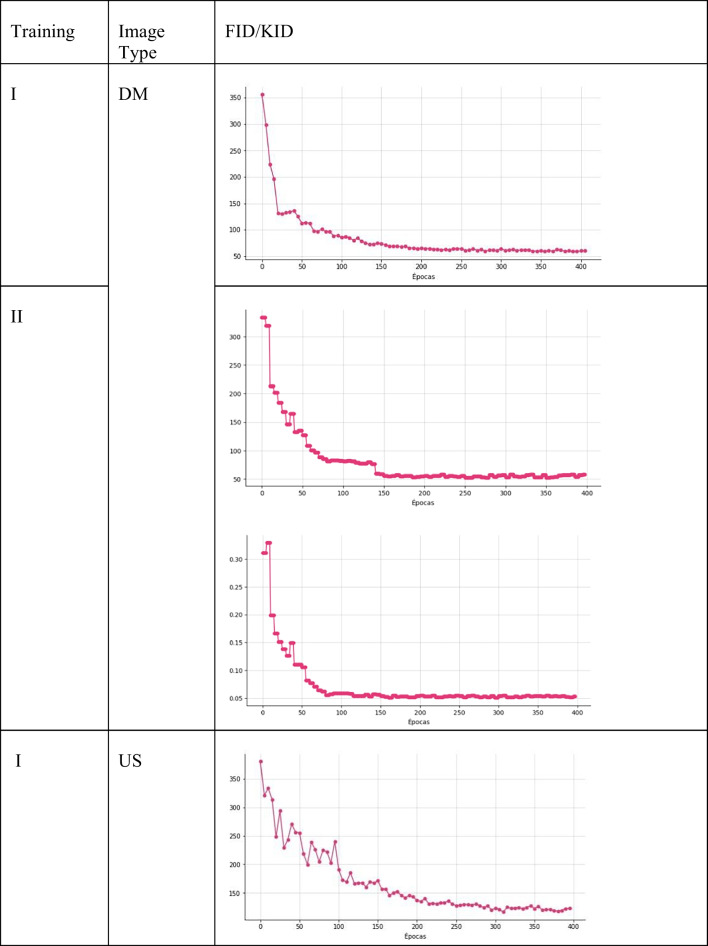
The FID and KID values a. SNGAN-FID values using mammography images during training I and II. b. SNGAN-FID values taken from US images

**Table 10:**
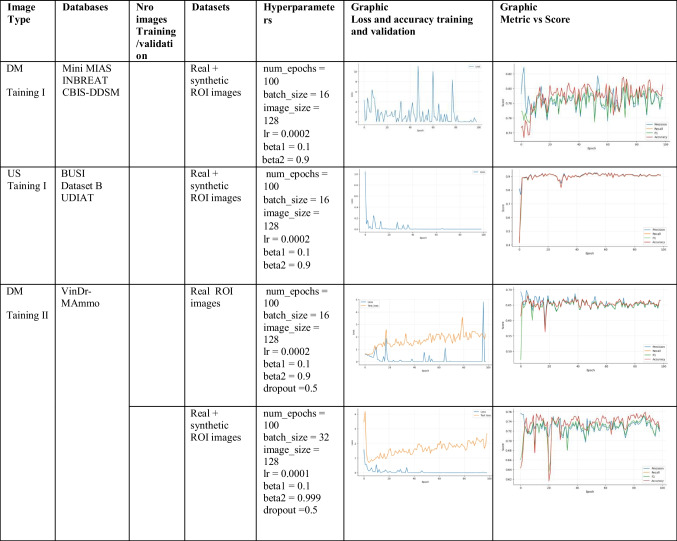
ResNet 18 evaluation model on training and validation datasets

## Data Availability

All codes are available as Mendeley Data: https://data.mendeley.com/preview/jh9trvbjbv?a=57b040ca-ae6d-4ebb-bc04-ac8c27deae59 [85].

## Appendix 1

Figure 5

## Appendix 2

Table 9

## Appendix 3

Figure 6

## Appendix 4

Table 10
